# Metacognition as a Predictor of Improvements in Personality Disorders

**DOI:** 10.3389/fpsyg.2019.00170

**Published:** 2019-02-08

**Authors:** Antonino Carcione, Ilaria Riccardi, Elena Bilotta, Luigi Leone, Roberto Pedone, Laura Conti, Livia Colle, Donatella Fiore, Giuseppe Nicolò, Giovanni Pellecchia, Michele Procacci, Antonio Semerari

**Affiliations:** ^1^Third Centre of Cognitive Psychotherapy – Italian School of Cognitive Psychotherapy (SICC), Rome, Italy; ^2^Department of Social and Developmental Psychology, Sapienza University of Rome, Rome, Italy; ^3^Department of Psychology, University of Campania Luigi Vanvitelli, Caserta, Italy; ^4^Department of Psychology, University of Turin, Turin, Italy

**Keywords:** metacognition, mentalization, personality disorders, psychotherapeutic process, psychotherapy outcome

## Abstract

Personality Disorders (PDs) are particularly hard to treat and treatment drop-out rates are high. Several authors have agreed that psychotherapy is more successful when it focuses on the core of personality pathology. For this reason, therapists dealing with PDs need to understand the psychopathological variables that characterize this pathology and exactly what contributes to maintaining psychopathological processes. Moreover, several authors have noted that one key problem that characterizes all PDs is an impairment in understanding mental states – here termed metacognition – which could also be responsible for therapy failures. Unfortunately, a limited number of studies have investigated the role of mentalization in the process of change during psychotherapy. In this paper, we assume that poor metacognition corresponds to a core element of the general pathology of personality, impacts a series of clinical variables, generates symptoms and interpersonal problems, and causes treatment to be slower and less effective. We explored whether changes in metacognition predicted an improvement among different psychopathological variables characterizing PDs; 193 outpatients were treated at the Third Center of Cognitive Psychotherapy in Rome, Italy, and followed a structured path tailored for the different psychopathological variables that emerged from a comprehensive psychodiagnostic assessment that considered patients’ symptoms, metacognitive abilities, interpersonal relationships, personality psychopathology, and global functioning. The measurements were repeated after a year of treatment. The results showed that changes in metacognitive abilities predicted improvements in the analyzed variables.

## Introduction

Psychotherapists and psychiatrists agree that Personality Disorders (PDs) are particularly troublesome to treat. Although psychotherapy is considered to be the treatment of choice for all PDs (Verheul and Herbrink, 2007; Livesley, 2012; Bamelis et al., 2014), the rate of treatment being prematurely interrupted is high (McMurran et al., 2010; Barnicot et al., 2011; Swift and Greenberg, 2012; Gamache et al., 2018; Gülüm, 2018). Unfortunately, research largely focuses on the treatment of borderline personality disorder (BPD), which may be an unjustified bias, since individuals with BPD represent a minority of PD sufferers requiring treatment (Dimaggio et al., 2013b).

Systematic research on factors associated with premature treatment interruption has not yet produced conclusive results; however, it is well known that the drop-out rate in PDs is particularly high. In an accurate metanalysis conducted by Swift and Greenberg (2012), the general treatment drop-out rate of 19.7% increased to 25.6% in the case of PDs. Furthermore, McMurran et al. (2010) discovered that the median drop-out rate in PD patients was 37%, while in a recent study by Gamache et al. (2018) the drop-out rate amounted to 40.8%. These data suggest that it might be particularly relevant to study the therapeutic process when treating PDs, since this would help to identify the main factors underlying personality pathology that might need to be addressed during treatment. Studies have generally investigated large sets of several pre-treatment variables without focusing on specific variables selected for treatment prognosis prediction (Gamache et al., 2018). These observations call for a remarkable effort in analyzing the treatment process and understanding the possible mechanisms of change during psychotherapy.

Additionally, several authors have agreed that therapeutic intervention should be centered on aspects of general personality pathology shared in different PDs; among these, a reduced ability to understand the minds of others seems to be particularly relevant (Fonagy, 1991; Semerari et al., 2003, 2007, 2014, 2015; Bateman and Fonagy, 2004, 2009; Minzenberg et al., 2006; Dimaggio et al., 2007; Gullestad et al., 2013). Moreover, the DSM 5 stresses the key role of reflective abilities, since in Section III and establishes that in order to diagnose a PD, it is crucial to consider the evaluation of the functioning level of the individual’s personality through their capacity to (1) self-reflect, thus promoting a stable sense of self and self-directivity and (2) understand others’ minds in order to establish and maintain empathetic and good relationships (American Psychiatric Association [APA], 2013).

The ability of understanding mental states has different denominations, but in the field of PDs it is often termed “mentalization” (Bateman and Fonagy, 2004; Bouchard et al., 2008; Choi-Kain and Gunderson, 2008) or “metacognition” (Semerari et al., 2003, 2007; Dimaggio and Lysaker, 2010; Carcione et al., 2011). These two terms have been used in numerous studies as similar concepts, and there is a broad consensus indicating that they refer to almost the same psychological function (Bo et al., 2014; Semerari et al., 2014; Fonagy and Bateman, 2016).

In this paper, we use the term *metacognition* to refer to a set of abilities that are crucial to: (1) identify mental states and ascribe them to oneself and others on the basis of facial expressions, somatic states, behaviors, and actions; (2) reflect and reason on mental states; (3) use information about mental states to make decisions, solve problems or for psychological and interpersonal conflicts and to cope with subjective suffering (Carcione et al., 2010, unpublished).

Only a limited number of studies have investigated the role of different metacognition abilities in the process of change during psychotherapy (Levy et al., 2006; Vermote et al., 2010; Maillard et al., 2017). Some studies have provided data about the role of mentalization as a moderator of the clinical outcomes of psychotherapeutic treatment for PDs (Gullestad et al., 2013). Other studies have investigated the predictive role of a series of constructs related to metacognition, such as psychological mindedness (PM; Appelbaum, 1973; Conte et al., 1990; McCallum et al., 2003; Ogrodniczuk et al., 2011), alexithymia (Nemiah and Sifneos, 1970), especially in creating major difficulties in identifying the aim of treatment, (Leweke et al., 2009; Ogrodniczuk et al., 2010; Nicolò et al., 2011) and affect-consciousness (AC – Monsen and Monsen, 1999), whose high pre-treatment levels predict improvements in Cluster C pathology (Gude et al., 2001).

Within this framework, in this study we assumed that poor metacognition corresponded to a core element of the general pathology of personality, the functioning of which impacts a series of clinical variables (and treatment). Therefore, we expected that improvements in metacognition would be associated with improvements in personality pathology.

We explored changes in metacognition and in a series of clinical variables (i.e., personality dysfunction, symptom distress, and interpersonal and psychosocial functioning) in a sample of patients treated for 1 year with a treatment specifically structured to improve metacognition (i.e., the Metacognitive Interpersonal Therapy, MIT; Semerari, 1999; Dimaggio et al., 2007, 2011; Fiore et al., 2008; Dimaggio et al., 2015; Carcione et al., 2016).

To comprehensively evaluate the changes of all the considered variables, we firstly compared the mean scores at the beginning of the treatment (T0) and after 1 year (T1). We expected that the mean scores of personality severity (the number of dysfunctional traits), symptom distress and interpersonal problems would decrease, while global functioning and metacognition would increase after 1 year of treatment. Secondly, we compared the associations between metacognition and the clinical variables considered at the beginning of the study and after 1 year of treatment. If low metacognition is a variable that could be conceived of as a core aspect across different PDs, then an improvement in metacognition should predict a reduction in personality pathology. Specifically, considering that poor metacognition is related to the severity of personality pathology measured through the number of PD criteria (according to the DSM IV-TR; Dimaggio et al., 2013a; Semerari et al., 2014), we hypothesized that improvements in metacognition are associated with improvements in personality pathology (i.e., a reduction in the number of dysfunctional traits). Furthermore, considering that an understanding of mental states is a requirement to regulate and master those same states (Carcione et al., 2011), we also hypothesized that an increase in metacognition is associated with a reduction in symptom distress among patients with PDs. Since it is also assumed that understanding the mental states of oneself and others is fundamental to the regulation of interpersonal relationships and helps individuals to overcome interpersonal problems (Dimaggio et al., 2007), our third hypothesis was that an increase in metacognition would be associated with a decrease in interpersonal problems. Additionally, we expected that improvements in metacognition were associated with improvements in global psychosocial functioning. We tested the hypotheses of an association between changes in metacognition and changes in all of the above considered clinical variables after 1 year of treatment through the use of a structural equation model with latent variables.

## Materials and Methods

### Participants

The sample consisted of 193 individuals who completed a 1-year treatment schedule in an Italian outpatient clinic between 2011 and 2017. The mean age of the sample was 33.37 years (SD = 9.54), ranging from 18 to 65. 59 participants (44.7%) were male and 73 (55.3%) were female. All participants met DSM-IV-TR (American Psychiatric Association [APA], 2000) diagnostic criteria for PD; DSM-IV Axis I diagnoses were assessed using the Structured Clinical Interview for the DSM-IV, Axis I Disorders (SCID-I; First et al., 1996). The inclusion criteria embraced patients with at least one PD (including those with histories of suicidal attempts or self-harm). On the other hand, the exclusion criteria were substance dependence, psychotic disorders, bipolar I disorder, delirium, dementia, mental retardation, severe medical conditions which precluded psychiatric medications, and medical conditions requiring hospitalization. Individuals who were enrolled in the study provided written informed consent.

Table 1 illustrates the demographic and diagnostic characteristics of the study sample and the percentage of PD diagnoses.

**Table 1:** Sample description.

N			Gender				Age M(SD)		
193			83 M (43%)				32.9 (10.1)		
			110 F (57%)

**Percentage of Diagnosis for PDs**										

**AV**	**DEP**	**OBS**	**PA**	**DE**	**PAR**	**ST**	**HIS**	**NAR**	**BDL**	**AS**

7.3	10.4	23.8	10.4	10.4	5.2	0.5	2.6	7.8	14.4	1.6

*M, Male; F, Female; AV, Avoidant PD; DEP, Dependent PD; OBS, Obsessive PD; PA, Passive-Aggressive PD; DE, Depressive PD; PAR, Paranoid PD; ST, Schizotypal PD; HIS, Histrionic PD; NAR, Narcissistic PD; BDL, Borderline PD; AS, Antisocial PD*.

### Measures

The *Structured Clinical Interview for DSM-IV* (SCID-II; First et al., 1997) was used to obtain diagnostic Axis-II profiles on the basis of the criteria of the DSM-IV (American Psychiatric Association [APA], 2000), which yielded 11 different categories of PD diagnoses. In this study, satisfactory inter-rater reliability was found in the application of the SCID-II. 20 SCID-II were rated twice; the internal consistency of the PDs traits ranged from 0.71 to 0.89 for the majority of the PD diagnoses; only four PDs (obsessive-compulsive, dependent, schizotypal, and passive-aggressive) achieved alphas above 0.60. The inter-rater reliability was adequate for both trait scores (a two-way mixed absolute agreement model for the Intraclass Correlation Coefficients (ICC) ranged from 0.87 to 0.99, mean = 0.94) and categorical diagnoses (average κ = 0.89).

The *Symptom Checklist-90-R* (SCL-90-R; Derogatis, 1977; α = 0.96) is a 90-item self-report inventory designed to reflect the psychological symptom patterns of psychiatric and medical patients. It is a measure of the current (state) psychological symptom status of a patient. The SCL-90-R measures nine primary symptom dimensions and generates an estimate of global psychopathology, the Global Severity Index (GSI), which has been adopted in the current study as a measure of symptoms.

The *Inventory of Interpersonal Problems*-47 (IIP-47; Italian version Ubbiali et al., 2011; α = 0.93) is a 47-item self-report scale which assesses interpersonal problems, and consists of five subscales: Interpersonal Sensitivity, Interpersonal Ambivalence, Aggression, Need for Social Approval, and Lack of Sociability.

The *Global Assessment of Functioning* (GAF; American Psychiatric Association [APA], 2000) is a valid measure of social functioning and is currently placed on the fifth axis of the DSM-IV-TR. It has shown reasonable psychometric properties (inter-rater reliability of approximately 0.80; Dworkin et al., 1990). For this study, the inter-rater agreement was good (ICC, *r* = 0.80, *p* < 0.001).

*The Metacognition Assessment Interview* (MAI). The MAI (Semerari et al., 2012; Pellecchia et al., 2015) is a semi-structured clinical interview designed to elicit and evaluate the metacognitive abilities of the participant during a brief narrative of a psychologically significant experience or event. During the interview, the participant is requested to describe the most troubling interpersonal experience they had experienced in the previous 6 months, a time frame selected in order to facilitate recall and to permit test-retest, avoiding recall biases, in the evaluation of changes during psychotherapy. The reported experience must be autobiographical, personal and involve another person, so that the individual’s ability to understand the mental state of others can be evaluated. Once the description of the episode is completed, the interviewer asks a list of questions, divided into four modules, to elicit and evaluate the 16 basic facets constituting metacognitive sub-functions (four facets are allocated to each sub-function). The interviewer assigns each of the 16 basic facets a score ranging from 1 to 5 using a Likert scale. The metacognitive functions assessed by the MAI are: Monitoring (MON), Integration (INT), Differentiation (DIF), Decentration (DEC), and Global score. MON is the ability to identify and label the components of our mental states in terms of emotions, thoughts, motivations and desires. People who can effectively monitor find it easy to give appropriate answers to questions such as “What do you think?” and “How do you feel?”. Impairments of this function compromise both the individual’s ability to describe his/her internal state and their ability to explain the reasons and motivations underlying his/her behavior. INT refers to the more general capacity of individuals to reflect upon different mental states and identify internal contradictions, conflicts and patterns. This metacognitive function allows us to adaptively organize mental content in terms of significance and subjective priority and thus to maintain behavioral coherence. An INT disorder causes mental processes and behaviors to be contradictory and unstable. DIF indicates the individual’s ability to recognize the representational nature of their mental states, distinguishing clearly between the internal psychological content and external reality. In the presence of impaired differentiation, imagination takes on the properties of the real world. In this perspective, if the patient is unable to recognize the subjectivity of his/her mental representations, he/she is also unable to maintain a critical distance from his/her own representations. DEC refers to the ability to assume other people’s perspectives and to make plausible hypotheses about their mental states. Specifically, it means being able to reflect on others’ intentions, thoughts and desires, independently of one’s own personal point of view.

The MAI was tested in two preliminary studies. In the first study, factor analysis was used to investigate 175 non-clinical subjects and revealed the presence of two higher order domains, which can be described, respectively, as the awareness of one’s own mental state and the awareness of others’ mental states (Semerari et al., 2012). In the second study, conducted with the same sample as this study, factor analysis indicated four factors, consistent with the structure of the MAI sub-functions, but which also confirmed the higher “two factor” structures identified in the first study (Pellecchia et al., 2015). Additionally, this study demonstrated a significant association between the MAI and alexithymia measured with Toronto Alexithymia Scale (TAS-20) (Bagby et al., 1994). In particular, MON scores and MAI global scores were associated with all TAS-20 dimensions and total scores (with correlation coefficients ranging from 0.24 to 0.39, *p* < 0.01). Moreover, MAI sub-functions and global scores resulted in an association with the global evaluation of interpersonal problems measured with the IIP-47 (Pilkonis et al., 1996), with a correlation coefficient ranging from 0.19 to 0.27 (*p* < 0.01).

In the present study, the MAI was administered and scored by three senior interviewers blind to the clinical diagnosis of the participants. A preliminary inter-rater reliability evaluation was carried out on 20 interviews. The ICC was used to estimate the correlation for every single function rated by different judges. A two-way mixed absolute agreement model was applied to conduct the ICC for each dimension of the MAI. The ICC for the MAI’s functions ranged from 0.55 to 0.72 for MON; from 0.50 to 0.67 for INT; from 0.49 to 78 for DIF; and from 0.45 to 0.61 for DEC; all analyses were significant (p < 0.001) and provided good inter-rater reliability. The internal consistency of the MAI dimensions was estimated with Cronbach’s alpha, which ranged from 0.85 to 0.89.

### The Treatment: Metacognitive Interpersonal Therapy (MIT)

The Metacognitive Interpersonal Therapy (MIT is an integrated approach, developed by the Third Center of Cognitive Psychotherapy in Rome, to treat PDs (Carcione et al., 2016). It aims to improve metacognitive abilities and to master problematic mental states. This treatment model derives from a) the analysis of clinical and research literature on PDs and (2) intensive research investigating the therapeutic process starting from a descriptive model of psychopathological functioning (Dimaggio et al., 2007, 2015; Semerari et al., 2014).

Metacognitive interpersonal therapy was developed within the framework of CBT, but it integrates the different procedures and techniques developed, even from a non-CBT approach (i.e., Mentalization Based Treatment-MBT, Dialectical Behavior Therapy-DBT), for the treatment of PDs. In particular, MIT shares with MBT the constant attention and focus on the patient’s reflective abilities and their efforts to increase these abilities as its principal aim.

Metacognitive interpersonal therapy can be schematically divided into five phases focused on different metacognitive functions:

(1) In the first phase, the principal aim is to develop the patient’s ability to monitor problematic states. The therapist attempts to make the patient aware of (a) the primary emotion, which is the basis of these states and (b) the intentions, motivations and goals underlying the most dangerous behaviors for the patient and which prevent a good therapeutic alliance from developing.
(2) The aim of the second phase is to develop an integrated view (i.e., the INT ability) of the current trends in the patient’s mental state. The therapist tries to: (a) focus on the transition of the states; (b) highlight the contradictions and conflicts and (c) reconstruct the modifications of the problematic states in conjunction with the patient. The awareness of the dynamics of the states is the basis for the greater tolerance of suffering which itself is increased using mindfulness and experiential techniques.
(3) The third phase is focused on the patient’s ability to consider the representational nature of thoughts. The therapist uses CBT techniques to promote the patient’s differentiation abilities, helping them to distinguish between representation and reality and to consider the subjectivity of one’s own point of view. In these two phases, the mastery of problematic states is achieved through behavioral modifications using cognitive behavioral and DBT techniques.
(4) In the fourth phase, the aim is to increase the awareness of dysfunctional interpersonal cycles (according to Safran and Segal, 1990; Safran and Muran, 2000). The therapist has to (a) focus on the self and interpersonal schemas (self/other representations) and (b) promote differentiation and decentration abilities using cognitive therapy procedures.
(5) The aim of the fifth phase is to develop a sense of self-agency. The therapist helps the patient to build autobiographical continuity in which the troubles and how he/she coped with them emerge in a coherent narrative.

The therapist, throughout the duration of the psychotherapy, must, at the right moment, debate with the patient the behavioral and problem solving (i.e., mastery) strategies spontaneously adopted, encouraging those which are more adaptive to cope with distress and interpersonal problems.

In addition to individual therapy, MIT can also provide group intervention aimed at improving metacognition using psychoeducation and role-playing techniques, with particular attention paid to the impact of metacognition on relational aspects.

### Procedure

All measures were administered at baseline (pre-treatment) and after 1 year of treatment. SCL-90-R and IIP-47 were self-reported by the patients; GAF was reported by a clinician, SCID-II interviews and MAI at T0 and T1 were administered by a clinical team of psychologists and psychiatrists from the Third Center of Cognitive Psychotherapy in Rome, Italy. Each patient was rated by the same clinician at both T0 and T1.

The therapists were psychiatrists and psychologists, all trained in CBT, with an expertise in PDs and an experience ranging from 5 to 35 years. The sample comprises outpatients who sought the services of a private clinical center (Third Center of Cognitive Psychotherapy). Patients followed a thorough assessment procedure: first, patients are interviewed by a senior psychiatrist and psychotherapist (at least 20 years of experience); thus, several diagnostic and clinical tests are administered and a diagnosis is established; then, in a team meeting, the patient is assigned to a psychotherapist, taking into consideration the peculiarities of the specific case and the expertise of the psychotherapist in treating similar cases. The center’s organizational procedure includes weekly team meetings for the discussion of the most complex cases, and to monitor the ongoing therapies.

The study was extensively explained to each participant, who signed a written consent form before entering into the study. Following the informed consent, all participants completed each of the self-report measures, and were then assessed during interviews. After the first evaluation (T0), participants were assigned to a therapist and attended the sessions every week for 1 year before being evaluated again (T1).

### Statistical Analyses

To test our hypotheses, the statistical analyses were divided into two phases. We firstly computed the number of SCID-II criteria met by each individual participating in the study; the resulting score was considered to be a global measure of the severity of personality pathology. An analysis of internal consistency supported the view that a general severity composite may be represented this way (at T0 α = 0.74; at T1 α = 0.88; Hopwood et al., 2011; Semerari et al., 2014).

During the first phase, a series of repeated ANOVA measures were computed in order to evaluate changes on all measures between early and late treatment. All results were evaluated against Holm’s sequential Bonferroni correction (Holm, 1979), and adjustments to alpha values were made to protect against inflated family-wise error rates.

**Figure 1:**
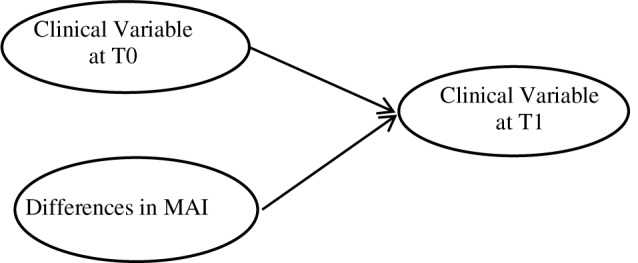
Conceptual diagram of the model.

Secondly, to investigate the role of metacognition in predicting changes in the severity of clinical variables we specified a structural equation model with latent variables, conceptually summarized in Figure 1. We modeled a latent criterion (or dependent variable), here termed “*Clinical Variable*,”, that summarized the observed variables relating to an array of clinical indicators (SCL90R-GSI, IIP-47, SCID II criteria, and GAF scores) at T1 (i.e., after 1 year of treatment). The latent predictor of improvements in metacognition was then linked to *Clinical Variable* at T1. To control for spurious effects, a latent variable *Clinical Variable* at T0 (using the same indicators as in T1) was also specified and linked with *Clinical Variable* at T1. Thus, any effect for metacognition on the T1 *Clinical Variable* cannot be traced back to spurious associations through *Clinical Variable* at T0. We expected that, over and above the association between *Clinical Variable* across the T0 and T1 time-lag, improvements in metacognition would be negatively associated (i.e., decrease) with the level of *Clinical Variable* at T1. A statistical analysis of the data was performed using SPSS 20.0 and LISREL 8.8.

## Results

### Changes Between Early and Late Treatment on Clinical and Functioning Measures

Table 2 presents the means and standard deviations for both outcome and predictor variables at T0 (the beginning of the treatment) and T1 (1 year later). Additionally, Table 2 summarizes the results for the repeated measures ANOVA for each variable. At T0, data showed generally high levels of severity and distress, and low scores of general functioning. At T1, the means showed significant changes compared with T0, indicating a general improvement in personality severity, symptom distress and levels of psychosocial and interpersonal functioning. Turning to levels of metacognition, a significant improvement was observed from T0 to T1. Such an improvement was detectable in both the MAI Global Score and the four MAI facets (Table 2).

**Table 2:** Outcome and predictor measure changes between early and late treatment (Repeated-measures ANOVA results of included variables).

Measures	Early Mean (SD)	Late Mean (SD)	Mean Difference	Fs	Partial Eta Squared
PD Severity	16.30 (6.59)	9.34 (6.39)	6.96	F(1,190) = 291.49^∗∗^	0.61
GSI	1.38 (0.59)	0.83 (0.55)	0.55	F(1,182) = 184.74^∗∗^	0.50
IIP-47	1.74 (0.65)	1.35 (0.64)	0.40	F(1,184) = 80.57^∗∗^	0.31
GAF	65.55 (10.15)	75.36 (11.41)	-9.81	F(1,149) = 138.50^∗∗^	0.48
**MAI**					
Monitoring	12.37 (2.71)	14.51 (2.12)	-2.14	F(1,182) = 122.86^∗∗^	0.41
Integration	11.13 (2.52)	13.21 (2.08)	-2.08	F(1,182) = 121.20^∗∗^	0.40
Differentiation	10.92 (2.74)	13.39 (1.93)	-2.47	F(1,182) = 146.43^∗∗^	0.45
Decentration	10.91 (2.73)	12.81 (2.38)	-1.90	F(1,182) = 103.23^∗∗^	0.36
Total score	45.33 (9.26)	53.92 (7.19)	-8.59	F(1,182) = 182.10^∗∗^	0.50

*PD Severity, number of criteria met at the SCID-II (*N* = 191); GSI, SCL90-R Global Severity Index (*N* = 183); IIP-47, Inventory of Interpersonal Problems (*N* = 185); GAF, Global Assessment of Functioning (*N* = 150); MAI, Metacognition Assessment Interview (*N* = 183). ^∗∗^*p* < 0.001*.

### Changes in Metacognition and Clinical Variables

**Figure 2:**
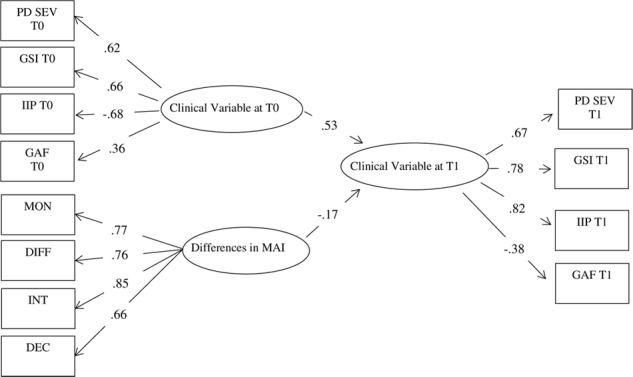
Structural equation model: Parameter estimates. Note: All parameters are significant. GSI, SCL90-R-Global Severity Index; IIP, Inventory of Interpersonal Problems; GAF, Global Assessment of Functioning; PD SEV, number of criteria met at the SCID-II; MON, Monitoring; DIFF, Differentiation; INT, Integration; DEC, Decentration; MAI, Metacognition Assessment Interview.

We tested the structural equation model with the latent variables depicted in Figure 2 (the obtained parameter estimates are summarized in the figure). A latent factor indexed by the observed scores at T1 (i.e., after 1 year of treatment) in the PD severity scores (the number of PD criteria met during SCID II) and also in the SCL90R-GSI scores, interpersonal problems (IIP-47) and GAF scores played the role of the dependent (or endogenous) variable. This *Clinical Variable* at T1 latent factor was predicted in the model by two independent latent factors. A first predictor, which mainly played a control role, was a latent factor *Clinical Variable* at T0, indexed by the PD severity scores, SCL90R-GSI, IIP-47 and GAF scores measured at T0 (at the beginning of the treatment). The second latent predictor was a “*Change in Metacognition*” factor, indexed by four difference-score indicators (T1-T0), one for each metacognition facet of the MAI. Utilizing the latent variables enables the study to more accurately predict the regression coefficients (because the measurement error is explicitly modeled and does not attenuate regression parameter estimates). This confirmative model also allows the testing of the global fit in terms of the ability of the model parameters to reproduce the observed data (Bollen, 1989). A Maximum Likelihood estimation was used to obtain the parameter estimates and standard errors. The ability of the model to reproduce the data is directly evaluated by a chi-square statistic; however, the chi-square statistic is excessively restrictive for large samples (Bollen, 1989), and therefore we would also evaluate the model fit by assessing the root mean square error of approximation (RMSEA), the comparative fit index (CFI), the non-normed fit index (NNFI), and the standardized root mean square residual (SRMR), as suggested by Hu and Bentler (1999). These latter indices are generally unaffected by sample size and provide a more comprehensive view of the model fit.

The model fitted the data satisfactorily. Although the chi-square statistic was significant [χ^2^(47, *N* = 218) = 84.11, *p* = 0.0007], the other fit indexes pointed to a reasonable fit: RMSEA = 0.060 [90% C.I. 0.039; 0.081]; CFI = 0.97; NNFI = 0.96; SRMR = 0.059. The RMSEA value was statistically undistinguishable from the so-called “close fit” hypothesis (RMSEA = 0.05), indicating negligible deviations in the reproduced data. CFI and NFFI were above the threshold of 0.95 generally associated with good fit, and the SRMR was below 0.08 (Hu and Bentler, 1999). The model appeared therefore fairly satisfactory.

Figure 2 summarizes the main parameter estimates. The measurement models (factor loadings) demonstrated satisfactory values, with significant loadings with the expected sign. Turning to the structural parameter estimates linking the latent variables, as should be expected, *Clinical Variable* at T0 were significantly and strongly linked with *Clinical Variable* at T1. Interestingly, once the cross-lag association between *Clinical Variable* across T0 and T1 was controlled for, increases in the metacognition scores were associated with decreases in *Clinical Variable* at T1.

## Discussion

Our study entered into the research field surrounding the existing relations between metacognition and its role in the outcome of treatment for PDs. Our purpose was to measure the specific functions of metacognition, their changes during treatment and their role as a predictor of personality severity changes and other outcome measures.

This study does not aim to assess the effectiveness of a specific treatment. Nevertheless, we believe that, given the lack of a broad range of sensitive measures of cognitive and affective dysfunctions found in PDs (Luyten et al., 2012; Chiesa and Fonagy, 2014), our study could add empirical evidence about the role of specific variables that are important to reduce therapeutic failures.

We firstly evaluated the mean differences in personality severity, symptom distress, interpersonal problems, global functioning, and metacognition both at the beginning of a treatment based on metacognition (MIT) and after 1 year of treatment. The results showed a general improvement in all the variables considered.

In the second hypothesis, we supposed that those improvements could be predicted by improved metacognition functioning developed by the patient during 1 year of MIT treatment. The results appeared consistent with these expectations.

These results can be discussed from two points of view: what they indicate with respect to the pathology of the individual’s personality, and what they indicate with respect to the psychotherapeutic process of PD patients.

From the point of view of personality pathology, if the initial general hypothesis that low metacognition is one of the general factors underlying this pathology is true, a metacognitive improvement would consequently be associated with a general improvement in the clinical variables associated with the disorder. Our data, through a structural equation model with latent variables, lent support to this argument, constituting indirect support to the central role played by low metacognition in PDs. The reported results are consistent with previous findings (Semerari et al., 2003, 2007, 2014, 2015; Bateman and Fonagy, 2004; Minzenberg et al., 2006; Dimaggio et al., 2007; Gullestad et al., 2013) that considered difficulties in understanding one’s own and others’ minds as core aspects of PDs. For example, Herpertz and Bertsch (2014) considered impairments in social cognition (i.e., facial emotion recognition, cognitive and emotional empathy, and theory of mind) to be a core concept that characterizes PDs. Other authors (Antonsen et al., 2016; Hayden et al., 2018) found an association between Reflective Function (RF) and the intensity of symptom distress and psychosocial impairment. Semerari et al. (2014) supported evidence that (1) metacognition is specifically impaired in PDs if compared to a clinical sample of non-PD patients, (2) the dysfunction is significantly correlated to the severity of personality pathology (measured as the number of criteria met in the SCID II) and (3) difficulties in metacognition are specific to different PDs (Semerari et al., 2007), for example in BPD (Semerari et al., 2005, 2015) and in Avoidant PD (AvPD) patients Pellecchia et al. (2018).

Furthermore, Pellecchia et al. (2018) compared patients with Social Phobia (SP), with AvPD, with both AvPD and SP and with other PDs without SP or AvPD criteria on metacognitive abilities, interpersonal functioning and global symptomatic distress. They found that patients with AvPD and AvPD+SP groups demonstrate poorer metacognition compared with SP patients; moreover, no differences were found in metacognition capacity between the groups with an AvPD diagnosis (AvPD+SP and AvPD) and the PD group without an AvPD diagnosis, which is consistent with the notion that poor metacognitive functioning is an element that differentiates personality pathology from anxiety disorders.

From the point of view of the impact on psychotherapeutic treatment, our data support the hypothesis that an increase in metacognitive abilities is a factor of change in personality pathology. Similarly, Chiesa and Fonagy (2014) found the mediator role of mentalization between early adverse experiences and PD diagnoses and between adversity and psychiatric distress. Our results are consistent with Gullestad et al. (2013), which provided data about the role of mentalization in psychotherapeutic treatment for PDs, and with other studies that have investigated the predictive value of related concepts, like psychological mindedness (PM) (Appelbaum, 1973), alexithymia (Nemiah and Sifneos, 1970) and affect-consciousness (Monsen and Monsen, 1999). Higher pre-treatment levels of PM have been found to predict favorable outcomes (Conte et al., 1990; McCallum et al., 2003; Ogrodniczuk et al., 2011). Additionally, convergent evidence shows that alexithymia impacts treatment, for example by creating major difficulties in identifying treatment aims or in generating negative reactions in the therapists (Leweke et al., 2009; Ogrodniczuk et al., 2010; Nicolò et al., 2011). Finally, in a study that examined the relationship between Affect Consciousness (AC) and cluster C personality pathology, a high pre-treatment level of AC predicted a reduction in avoidant personality pathology, but not in dependent or obsessive-compulsive PD-traits. One exception is the data of Gude et al. (2001), where an increase in AC during therapy was not associated with improvements in personality pathology. This difference could be due to the fact that AC covers only some aspects of reflective abilities, as the ability to perceive and organize specific affects, while metacognition includes several other abilities in understanding one’s own and others’ minds (including not only emotional but also cognitive awareness). This difference could mean that metacognition, as measured through the MAI, clinically captures more relevant functioning.

Together, the data encourage the investigation of aspects of functioning underlying the various PDs and the refinement of the intervention focusing on these dimensions, in line with the suggestion of Bateman and Fonagy (2009).

### Limitations

The present study has a number of limitations that should be acknowledged. Firstly, data are mostly based on self-report measures (i.e., symptom distress and interpersonal problems). However, it should also be emphasized that the main variable of the present study (i.e., metacognition) was measured through a semi-structured interview, assuaging concerns of inflated associations due to common method biases. Moreover, other variables of interest, such as alexithymia levels, should be added in future studies concerning metacognition.

To obtain a fairly-sized sample, we did not distinguish among different PDs. Further studies could be extended in larger groups representing specific diagnoses. Nonetheless, our study was mainly concerned with the severity of personality functioning and distress, therefore our sample and methods appeared to be consistent with our research perspective. Moreover, we did not include a follow-up measurement, so we could not verify whether the improvement we depicted would change or remain constant after 1 year of treatment.

Finally, the design of our study was not aimed at testing the effectiveness of MIT treatment on PDs or to measure the drop-out rate. What our study does provide is corroborating evidence that improvements in metacognitive abilities go hand-in-hand with improvements in the severity of personality pathology and its associated symptoms. Future research should consist of clinical trials in order to determine whether a possible causal relationship exists between improvements in metacognition and a series of outcome variables, and to test if a psychotherapy for PDs focused on metacognition would actually reduce the drop-out rate compared with other psychotherapies.

## Conclusion

Our data supported the hypothesis that changes in metacognitive functioning would explain a significant portion of personality pathology, together with an improvement in symptoms and interpersonal and social functioning after 1 year of treatment. The reduction in distress levels can be explained by the fact that metacognition abilities might increase individuals’ ability to cope with mental states as a source of subjective suffering, showing that the metacognition construct is able to capture clinically relevant phenomena.

## Ethics Statement

The protocol was approved by the Scientific and Research Ethic Committee at School of Cognitive Psychotherapy, Rome, Italy. All participants signed written consent forms before participating in the study.

## Author Contributions

AC, IR, and AS conceived the study. EB, IR, and RP curated the data. EB and LL performed the formal analysis. AC, IR, EB, and AS investigated the study. AC, GN, MP, and AS administered the project. LaC, LiC, DF, and GP contributed to resources. AC and AS supervised the study. AC, EB, IR, and AS wrote the original draft of the manuscript.

## Conflict of Interest Statement

The authors declare that the research was conducted in the absence of any commercial or financial relationships that could be construed as a potential conflict of interest.
